# The Relationship between Illness Perception and Self-Care Behaviors among Hemodialysis Patients

**Published:** 2020-04

**Authors:** Mahnaz Rakhshan, Fatemeh Mirshekari, Fereshteh Dehghanrad

**Affiliations:** 1 Community Based Psychiatric Care Research Center, School of Nursing and Midwifery, Shiraz University of Medical Sciences, Shiraz, Iran.; 2 Student Research Committee, School of Nursing and Midwifery, Shiraz University of Medical Sciences, Shiraz, Iran.; 3 Nursing Department, School of Nursing and Midwifery, Shiraz University of Medical Sciences, Shiraz, Iran.

**Keywords:** *Hemodialysis Patients*, *Illness Perception*, *Self-Care Behaviors*

## Abstract

**Objective:** Illness perception and self-care behaviors can result in higher levels of health behavior among hemodialysis patients. The present study aimed to assess the relationship between illness perception and self-care behaviors among hemodialysis patients who referred to the hospitals affiliated to Shiraz University of Medical Sciences in 2017.

**Method** **:** In this descriptive cross sectional study, 216 hemodialysis patients who referred to the hemodialysis wards of the hospitals affiliated to Shiraz University of Medical Sciences were selected via convenience sampling. The study data were collected using a demographic information form, Brief Illness Perception Questionnaire (9 items), and a 15-item Self-care Behavior of Hemodialysis Patients Questionnaire. Then, the data were analyzed using independent t test and one-way ANOVA. Also, significance level was set at P < 0.05.

**Results: **The mean age of the study participants was 30.15+6.65 years. Also, most of the participants were female (n = 125, 58%). The results revealed a significant relationship between illness perception and self-care behaviors among hemodialysis patients. A significant relationship was observed between the following items: daily weight control and perception of consequences (r = 0.200, p = 0.001), between contacting the physician at the time of shortness of breath and consequences (r = 0.209, p = 0.001), between weight control according to the physician’s order and consequences (r = 0.763, p = 0.001), and between adherence to fluids restriction and identity(r = 0.149, p < 0.05).

**Conclusion: **Considering the relationship between illness perception and self-care, beliefs as illness perception have to be incorporated into self-care programs designed for hemodialysis patients so as to promote their self-care behaviors. These findings can be used for planning and implementing care for hemodialysis patients.

The daily increasing prevalence of chronic diseases is the most significant phenomenon encountered by health staff and communities in the 21st century (1). One of such chronic diseases is chronic renal failure, which can lead to progressive and irreversible decline of renal function (2). The global prevalence of chronic renal failure has been reported to be 242 per 1,000,000 population (3). Moreover, it has been estimated that nearly 39 000 individuals suffer from renal failure in Iran (4). 

When renal function reaches 10%-15% of its normal rate, alternative methods may be required. Hemodialysis is one of the most common alternative techniques used in this context (2). Hemodialysis has been defined as the process of filtering waste materials and fluids accumulated in the body due to renal failure (2). 

According to the reports provided by the Kidney Foundation of Iran, more than 15 000 individuals out of the 39 000 renal patients are being treated by hemodialysis. It should be noted that the global rate of hemodialysis is 3% (5). 

However, hemodialysis patients are not fully capable of eliminating their self-care behaviors deficiencies. Thus, survey in this respect is recommended (6). Self-care activities in dialysis patients can play a critical role in adaptation with disease process, quality of life improvement, reduction of number of hospitalizations, and length of hospital stay, reduction of treatment costs, and decrease of mortality (7). Indeed, a large body of evidence has indicated that patients suffering from chronic diseases who had great self-care abilities benefitted from high rates of self-efficacy as well (8). 

Some researchers also investigated the relationship between self-care and disease control ability in patients with chronic diseases. For health care providers, understanding how clients perceive their illness may be important in providing the best care. One method to assess the opinion of clients about their disease is via checking illness perceptions. In this regard, the results of Hyden et al (2009) study demonstrated that improvement of illness perception could be effective in promoting self-care behaviors (9). On the other hand, improving illness perception also played a positive role in disease management among patients with chronic disorders (10). In the same vein, Carrillo Algarra et al (2013) revealed that promotion of illness perception could affect the continuation of the treatment process and improvement of self-care behaviors in chronic diseases (12). 

Illness perception can be different depending on social and cultural circumstances. To the authors’ knowledge, no study has assessed the relationship between illness perception and self-care in hemodialysis patients. Therefore, the present study aimed to determine the relationship between illness perception and self-care behaviors among hemodialysis patients who referred to hospitals in Shiraz in 2017.

## Materials and Methods

In this descriptive-analytical study, the research population included all hemodialysis patients who referred to the educational hospitals affiliated to Shiraz University of Medical Sciences, the most comprehensive medical center in south of Iran. Participants were aware about the goals and method of the study. They were also reassured about the voluntary nature of the study. The sample size was estimated using the following formula:

n=(Z1-α 2+Z1-β)2(12Ln(1+r1-r)2+3

 A total of 216 patients were selected via convenience sampling. Inclusion criteria of the study were as follow: age above 18 years, willingness to participate, ability to read and write, and passage of at least 6 months from renal failure diagnosis. Exclusion criteria were death or withdrawal from the study.

The study data were collected using 3 questionnaires. The first one was a demographic information form designed based on literature review (13) and experts’ opinions. This form included questions about age, sex, education level, duration of the disease, body mass index (BMI), risk factors of the disease, and smoking status. 

The second data collection instrument was the Self-care Behavior of Hemodialysis Patients Questionnaire. The primary structure and items of the questionnaire were designed according to literature review and experts’ opinions. Then, the psychometric properties of the questionnaire were assessed to determine its validity (face and content validity) and reliability (internal consistency and stability). The face validity of the questionnaire was approved by 8 hemodialysis patients and nursing specialists. Then, its content validity was evaluated using Content Validity Ratio (CVR) and Content Validity Index (CVI). All items with CVR > 0.79 and CVI > 0.75, based on experts’ opinions, were retained. CVR and CVI were found to be 1 and 0.87, respectively. Moreover, the reliability of the questionnaire was confirmed by Cronbach’s alpha = 0.86 and internal consistency = 0.89. The final version of the questionnaire included 15 questions responded through a 5-point Likert scale with the minimum score of 1 and maximum of 5.

To assess illness perception, the Brief Illness Perception Questionnaire (BIPQ) was used. This questionnaire consisted of 9 items to explore the patients’ cognitive and emotional representations (19). In this questionnaire, 5 items assessed the patients’ cognitive responses, which included consequences, timeline, personal control, cure/medical control, and illness coherence. Furthermore, 2 items (concerns and emotions) evaluated emotional representations. One item explored the ability to understand the disease, and the last item was an open question asking patients to list 3 main reasons for their disease (20). Pacheco-Huergo et al (2012) reported the content validity of the questionnaire to be 0.86 and its reliability to be 0.91 (24). In Iran, the Cronbach’s alpha of the Persian version of the questionnaire was reported to be 0.84, indicating the appropriate reliability of the instrument (21). Considering the fact that this questionnaire had not been used in hemodialysis patients, its CVI and CVR were found to be 0.73 and 0.86, respectively in the present study. In addition, its reliability was approved by Cronbach’s alpha = 0.89 among 30 patients.

Moreover, to determine the relationship between illness perception and self-care behaviors among hemodialysis patients, Pearson’s correlation coefficient and regression analysis were used. All analyses were performed using the SPSS statistical software, version 18 (p < 0.05). Also, questionnaire distribution and data collection were done by a research assistant.

***Ethical Consideration***

The researchers presented themselves and the purpose of the research to the participants and assured them that all data recorded during the interview was kept confidential. Then, participants who wish to participate in the study were chosen. Participants were also assured that they would not be excluded from the interview process at any stage of the research. Other ethical considerations include: (1) acquire written consent from participants; (2) Participants were assured that they would be provided with the study results if they so desired; (3) adhering to ethical considerations regarding the confidentiality of research unit information; (4) acknowledging all those who cooperated in conducting the research; (5) obtaining permission from the Ethics Committee of Shiraz University of Medical Sciences (95-01-08-13053). 

## Results

This study was performed on 216 eligible hemodialysis patients. Of the patients, 125 (58%) were female. The mean age of the participants was 30.15+6.65 years (Table 1). 

The mean score of illness perception was higher in consequences (4.57), emotional representations (4.21), and cure/medical control (4.06) dimensions, but moderate and low in other ones (Table 2). Considering item 9, 36.36% of the participants stated that the physical disorder was the reason for their disease.

The mean score of self-care was 4.8 in weight control, 4.5 in taking a walk, and 3.9 in fluids restriction dimensions. The mean scores of other dimensions have been presented in Table 3.

The results revealed correlations between illness perception and self-care behaviors among hemodialysis patients. The highest correlations were observed between the following items: daily weight control and perception of disease consequences (r = 0.200, p =0.001), between contacting the physician at the time of shortness of breath and consequences (r = 0.209, p = 0.001), between weight control according to the physician’s order and consequences (r = 0.763, p = 0.001), and between adherence to fluids restriction and identity (r = 0.149, p < 0.05). Further results regarding correlations between illness perception dimensions and self-care behaviors are presented in Table 4.

**Table 1:** The Frequency Distribution and Percentage of the Participants Based on the Demographic Information Questionnaire

	**Sample**	**Frequency (Percentage %)**
Gender	Female	125(58%)
	Male	91(42%)
	Total	216(100%)
Education level	Primary school	101(46.8%)
	Middle school	51(23.6%)
	Diploma and A.D.	20(9.3%)
	Bachelor’s and higher	44(20.3%)
	Total	216(100%)
History of diabetes	Yes	126
	No	90
Cigarette smoking	Yes	109
	No	126
Disease duration	1-3 years	63
	3-5 years	52
	6-8 years	45
	8 years and higher	56
Age	Group	Mean
	Females	30.77
	Males	29.31

**Table 2:** BIPQ Dimension Mean Scores (Standard Deviation) in Hemodialysis Patients

**Components**	**Mean**	**SD**	**t**	**P-value**
Consequences	4.57	1.49	44.95	0.001
Timeline	3.59	1.86	28.34	0.001
Personal control	2.78	1.49	27.34	0.001
Cure/medical control	4.06	1.79	33.24	0.001
Identity	3.48	1.97	25.92	0.001
Concern	3.30	2.24	21.65	0.001
Coherence	2.97	2.14	20.35	0.001
Emotional representations	4.21	2.25	27.44	0.001

P-Value< 0.05

**Table 3:** Mean Scores (Standard Deviation) of Self-Care Behaviors in Hemodialysis Patients

	**Items**	**Mean ** **+** ** SD** ^*^
1	I weigh myself every day.	4.8+0.6
2	I call my doctor or the hemodialysis ward in case of shortness of breath.	2.6+0.9
3	I call my doctor or the hemodialysis ward in case of increased edema.	2.6+0.9
4	I try to maintain my weight according to doctor’s/nurse’s recommendations.	2.8+0.9
5	I adhere to fluids restriction.	3.9+1.3
6	I rest during the day.	2.5+1.3
7	I call my doctor in case of extreme fatigue.	2.1+0.7
8	I adhere to my food regimen according to doctor’s/nurse’s recommendations.	3.2+1.3
9	I consume medications according to the doctor’s orders.	1.9+0.9
10	I get influenza vaccine every year.	1.6+0.9
11	I try to have light walking programs during the week.	4.5+0.9
12	I am often careless about self-care.	4.7+0.6
13	I seek better ways of self-care.	1.8+0.9
14	I manage my deeds whenever needed.	2.5+0.8
15	I do the necessary diagnostic experiments routinely.	1.2+0.9
	Total score	2.5+0.40

**Table 4:** The Correlation between BIPQ Dimensions and Self-Care Behaviors in Hemodialysis Patients

		**Emotional ** **representations**	**Coherence**	**Concern**	**Identity**	**Medical ** **control**	**Personal ** **control**	**Timeline**	**Consequences**
I weigh myself every day.	Correlation coefficient	0.055	0.061	0.059	0.071	0.058	0.015	0.050	0.200**
	P-value	0.168	0.102	0.125	0.194	0.125	0.262	0.589	0.001
I call my doctor or the hemodialysis ward in case of shortness of breath.	Correlation coefficient	0.003	0.073	0.063	0.040	0.045	0.031	0.063	0.209**
	P-value	0.829	0.110	0.118	0.470	0.105	0.472	0.383	0.001
I call my doctor or the hemodialysis ward in case of increased edema.	Correlation coefficient	0.010	0.035	0.020	0.086	0.009	0.083	0.017	0.160*
	P-value	0.825	0.322	0.535	0.578	0.885	0.869	0.789	0.019
I try to maintain my weight according to doctor’s/nurse’s recommendations.	Correlation coefficient	0.054	0.073	0.056	0.088	0.060	0.133	0.117	0.763**
	P-value	0.762	0.777	0.478	0.424	0.188	0.789	0.489	0.001
I adhere to fluids restriction.	Correlation coefficient	0.080	0.021	0.107	0.149*	0.050	0.054	0.138*	0.702**
	P-value	0.125	0.219	0.229	0.032	0.507	0.323	0.047	0.001
I rest during the day.	Correlation coefficient	0.122	0.016	0.065	0.049	0.075	0.054	0.021	0.235**
	P-value	0.533	0.722	0.08	0.324	0.125	0.109	0.378	0.001
I call my doctor in case of extreme fatigue.	Correlation coefficient	0.085	0.056	0.047	0.050	0.011	0.075	0.107	0.214**
	P-value	0.896	0.224	0.229	0.219	0.727	0.769	0.701	0.001
									
I adhere to my food regimen according to doctor’s/nurse’s recommendations.	Correlation coefficient	0.032	0.133	0.051	0.131	0.097	0.266**	0.183**	0.158*
	P-value	0.688	0.824	0.183	0.563	0.783	0.001	0.001	0.031
I consume medications according to the doctor’s orders.	Correlation coefficient	0.045	0.000	0.134*	0.058	0.014	0.272**	0.138*	0.127
	P-value	0.08	0.524	0.125	0.569	0.789	0.001	0.047	0.338
I get influenza vaccine every year.	Correlation coefficient	0.027	0.049	0.112	0.013	0.047	0.208**	0.112	0.129
	P-value	0.72	0.323	0.125	0.569	0.789	0.001	0.569	0.689
I try to have light walking programs during the week.	Correlation coefficient	0.118	0.041	0.058	0.004	0.086	0.239**	0.197**	0.067
	P-value	0.38	0.524	0.125	0.569	0.789	0.001	0.001	0.178
I am often careless about self-care.	Correlation coefficient	0.050	0.052	-0.077	-0.083	-0.037	-0.302**	-0.191**	0.078
	P-value	0.662	0.756	0.635	0.663	0.578	0.001	0.001	0.102
I seek for better ways of self-care.	Correlation coefficient	0.042	0.068	0.025	0.024	0.007	0.145*	0.096	0.029
	P-value	0.423	0.578	0.423	0.123	0.968	0.038	0.189	0.268
I manage my deeds whenever needed.	Correlation coefficient	0.085	0.167*	0.099	0.072	0.166*	-0.135*	-0.234**	0.092
	P-value	0.745	0.020	0.869	0.569	0.019	0.02	0.001	0.833
I do the necessary diagnostic experiments routinely.	Correlation coefficient	-0.154*	0.204**	0.137*	0.109	-0.123	0.205**	0.171*	0.098
	P-value	0.031	0.001	0.045	0.758	0.365	0.001	0.015	0.836

* P-Value< 0.05

** P-Value< 0.001

## Discussion

This study aimed to investigate the relationship between illness perception and self-care behaviors among hemodialysis patients. The results revealed a significant relationship between illness perception and self-care behaviors. Moreover, the patients believed that the disease had a slight effect on their lives. Also, they perceived the disease to have a short timeline; in other words, they considered the disease to be acute. In the same line, Jahanbeen et al concluded that hemodialysis patients in Fasa, in Fars province of Iran had a moderate perception of the disease (25). Considering the 6 dimensions of illness perception, these patients should be helped to gain a better perception of their disease.

In the present study, the patients obtained a moderate self-care score. Khoshtarash also conducted a study in 2013 and reported moderate self-care among diabetic patients (17). Hwin performed a research in 2011 and indicated that half of diabetic patients adhered moderately to their self-care behaviors (26). It seems that patients’ adherence to their self-care behaviors is the outcome of a complex process requiring the identification of various dimensions and empowerment of facilitating factors.

In the present study, the patients obtained a moderate score in adherence to their medication regimens. In 2012, Nieuwenhuis demonstrated that although all patients had reported the consumption of the prescribed medications, direct monitoring revealed that only 76% adhered to their medication regimens completely (27). In contrast, Khoshtarash in 2013 (17) and Abootalebi in 2011 (18) indicated that adherence to physicians’ medication orders were among the best self-care behaviors in patients with heart diseases. The discrepancy observed in the results may be attributed to differences in research populations and methodologies. Nonadherence to treatment regimen may result from lack of knowledge about the disease process and outcomes. Thus, self-care behaviors can be promoted by educational methods based on patient empowerment, which are performed via cooperation of patients and their family members. Conducting studies to identify the perceived barriers against self-care behaviors can also be helpful in this regard.

The findings of the present study showed a high level of adherence to nutrition regimen among the patients. However, the results of the research by Frediani in 2012 demonstrated that only 33% of hypertensive patients adhered to the low-sodium diet (29). The difference between the results may be due to variations in the research populations and methodologies. Considering the importance of adherence to diet in improving the disease process and reducing the serious complications of chronic disorders (30), studies and continuous educational programs should be conducted to improve the knowledge levels and health behaviors of the public.

In the present study, the patients weighed themselves every day. Also, the results showed moderate weight maintenance based on physician’s/nurse’s advice. In the study performed by Khoshtarash in 2013, the lowest adherence to self-care behaviors was related to daily weighing among patients with heart diseases (17). Although daily weighing is among self-care programs recommended for patients with chronic diseases, it is rarely performed even by patients with severe symptoms.

The present study revealed no significant relationships between demographic characteristics and illness perception and self-care behaviors. In other words, no significant differences were observed among the patients in different age and gender groups with various education levels with respect to illness perception and self-care behaviors. The results of this study were in agreement with those of the study conducted by Abootalebi (18) on 72 patients with heart failure in which no significant relationship was found between residential status and gender with self-care agency.

## Limitation

One of the study limitations was investigation of the patients in the treatment centers affiliated to one medical university. Hence, further studies are recommended to evaluate a larger sample of hemodialysis patients in several treatment centers affiliated to other universities of medical sciences. Furthermore, it is suggested that future studies be conducted on the impact of self-care programs developed for these patients.

## Conclusion

Considering the relationship between illness perception and self-care, beliefs as illness perception are recommended to be incorporated into self-care programs designed for hemodialysis patients to improve their self-care behaviors. In this regard, nurses and other members of the health care team can use the findings of the present study to design and implement care plans for hemodialysis patients. 
